# The Social Media Index: Measuring the Impact of Emergency Medicine and Critical Care Websites

**DOI:** 10.5811/westjem.2015.1.24860

**Published:** 2015-03-17

**Authors:** Brent Thoma, Jason L. Sanders, Michelle Lin, Quinten S. Paterson, Jordon Steeg, Teresa M. Chan

**Affiliations:** * Learning Laboratory and Division of Medical Simulation, Department of Emergency Medicine, Massachusetts General Hospital, Boston, Massachusetts; † University of Saskatchewan, Emergency Medicine, Saskatoon, Saskatchewan; ‡ MedEdLIFE Research Collaborative, San Francisco, California; § University of Pittsburgh, Department of Epidemiology, Pittsburgh, Pennsylvania; ¶ Harvard Affiliated Emergency Medicine Residency, Brigham and Women’s Hospital, Massachusetts General Hospital, Boston, Massachusetts; || University of California, San Francisco, Department of Emergency Medicine, San Francisco, California; # College of Medicine, University of Saskatchewan, Saskatoon, Saskatchewan; ** McMaster University, Division of Emergency Medicine, Department of Medicine, Hamilton, Ontario

## Abstract

**Introduction:**

The number of educational resources created for emergency medicine and critical care (EMCC) that incorporate social media has increased dramatically. With no way to assess their impact or quality, it is challenging for educators to receive scholarly credit and for learners to identify respected resources. The Social Media index (SMi) was developed to help address this.

**Methods:**

We used data from social media platforms (Google PageRanks, Alexa Ranks, Facebook Likes, Twitter Followers, and Google+ Followers) for EMCC blogs and podcasts to derive three normalized (ordinal, logarithmic, and raw) formulas. The most statistically robust formula was assessed for 1) temporal stability using repeated measures and website age, and 2) correlation with impact by applying it to EMCC journals and measuring the correlation with known journal impact metrics.

**Results:**

The logarithmic version of the SMi containing four metrics was the most statistically robust. It correlated significantly with website age (Spearman r=0.372; p<0.001) and repeated measures through seven months (r=0.929; p<0.001). When applied to EMCC journals, it correlated significantly with all impact metrics except number of articles published. The strongest correlations were seen with the Immediacy Index (r=0.609; p<0.001) and Article Influence Score (r=0.608; p<0.001).

**Conclusion:**

The SMi’s temporal stability and correlation with journal impact factors suggests that it may be a stable indicator of impact for medical education websites. Further study is needed to determine whether impact correlates with quality and how learners and educators can best utilize this tool.

## INTRODUCTION

The number of educational blogs and podcasts in emergency medicine and critical care (EMCC) has increased dramatically in the past decade,^1^ paralleling the growth of digital scholarship in other areas of science.^2^,^3^ This proliferation has led to difficulty finding high quality resources^2^,^4^ and assessing their scholarly value.^3^,^5^ If these problems are not addressed, early adopters could err due to the consumption of poor quality information, and educators could stop contributing due to a lack of recognition. Impact and quality assessment tools for these resources would help address both potential problems.

Unfortunately, minimal research has been done to date on how to critically appraise the quality of secondary resources in medical education. Blogs and podcasts could be viewed as the 21^st^ century equivalent of textbooks and lectures,^6^ but these historic parallels provide little guidance on quality assessment. Continuing medical education lectures do not typically undergo full peer review before presentation and printed textbooks have variable review processes. Solutions such as incorporating formal peer review processes into blogs and podcasts have been pioneered^7^ but have not been widely adopted.

New metrics are needed to assess the impact of blogs and podcasts in a similar way that impact factors assess journals. The journal impact factor (JIF) and Eigenfactor™ metrics were developed to illustrate the scientific importance of traditionally published academic literature.^8^–^11^ While never devised to be a marker of quality, “the use of the impact factor as a measure of quality is widespread because it fits well with the opinion we have in each field of the best journals in our specialty.”^11^,^12^ Despite arguments that impact factors are a poor surrogate for quality, they are used for university rankings and inform the hiring, funding, and promotion/tenure decisions that affect scholars.^11^,^13^ Regardless, the indices that are used for traditional journals cannot be applied to websites.

Alternative metrics (“altmetrics”) that assess online engagement through a broad range of measures have been found to correlate with the citations of journal articles,^14^ and are increasingly being recognized by institutions and granting organizations.^3^ Altmetrics from social media sources such as Twitter, Facebook, Google+, LinkedIn, and Reddit have been found to “crowd-source” impact assessment by combining individual endorsements.^15^ External composite rankings of website importance, popularity, and impact, such as Alexa Rank^16^ and Google PageRank,^17^ are metrics that use proprietary methods that incorporate website traffic and inbound/outbound links. Impact Story is a new, web-based tool that helps to quantify the impact of individual blog posts, datasets, and research articles for individual authors.^18^,^19^ While these novel metrics are potentially useful for assessing the impact of an individual blog post or podcast, they are unable to identify high-impact blogs and podcasts for learners and educators.

In this paper we propose and define the Social Media index (SMi), a new metric that combines various altmetrics to measure the impact of websites as a whole. It differs from the metrics previously described in that it combines social media followership with composite website rankings into a score for a website rather than an article, blog post, or journal. It was derived using open-access EMCC podcasts and blogs because of the large number of these resources available.^1^ In addition, we assessed the ability of the SMi to measure impact by calculating the SMi scores for EMCC journals and assessing their correlation with known journal impact metrics.

## METHODS

The SMi was developed by the lead author of this paper (BT). Pilot versions have previously been published on the emergency medicine blogs *BoringEM*^20^ and *Academic Life in Emergency Medicine*.^21^

### Website and Journal Inclusion Criteria

We obtained a list of 245 EMCC websites using a previously described methodology.^1^ A prospective, snowball sampling technique was used prospectively on an annual basis between 2002 and 2013 to compile a database of blog and podcast websites that were linked to each other. Additional websites were identified through personal communications, social media accounts, and a self-report form on the *Life in the Fast Lane* (http://lifeinthefastlane.com) website. We conducted a retrospective keyword search using Google in November 2013 using the terms: (“emergency medicine” OR “critical care” OR “intensive care”) AND (podcast OR blog) to identify any websites missed using the other processes. All websites found were reviewed and included in the study if they hosted freely accessible blogs or podcasts related to EMCC, were written in English, were active within the previous six months, and were not hosted on an institution’s or medical journal’s website.

Journal inclusion criteria were decided *a priori* to provide a broad range of literature of relevance to EMCC physicians. As categorized by the 2012 Journal Citation Report Journal Impact Factor,^22^ the top five “medicine, general & internal” journals (in order: *New England Journal of Medicine*, *Journal of the American Medical Association*, *Lancet*, *British Medical Journal*, and *PLOS Med*) and all “emergency medicine” and “critical care” journals composed in English were considered for inclusion. Journals with Facebook and Twitter accounts were included in the analysis.

### Variable Selection

The five variables described in Table 1 (Alexa Rank, Google PageRank, Twitter Followers, Facebook Likes, and Google+ Followers) were assessed to be components of the SMi. We considered these variables because they were publicly available metrics used by many EMCC websites. Personal or website accounts (whichever was greater) were eligible for Twitter Followers and Google+ Followers because a large number of websites are promoted on these platforms exclusively using openly accessible personal accounts. Only the Facebook pages of websites (rather than individuals) were eligible for inclusion because personal accounts are considered private.

### Data Collection

We gathered data on all five metrics from the included EMCC websites for four consecutive weeks between December 29, 2013 and January 19, 2014 and again on July 27, 2014. The final collection point was initially planned for six months; however, the authors were unavailable to collect data until nearly seven months. On each date, data for all websites were collected within a single 12-hour period by one of two authors (QP, JS) and audited by a third (BT). Data were gathered on the EMCC journals on January 20, 2014, within 24 hours of the website data collection on January 19, 2014.

### Deriving the Social Media Index

We initially calculated the SMi using raw data. However, due to high skewness, modified versions were calculated using logarithmically transformed data and ordinal data. In all formulas each of the five metrics was given equal weight by normalizing the individual values relative to the highest value. We then added the scores for each component to calculate the SMi.

### Analysis

The rankings of the SMi and each of its components were calculated separately for EMCC website and journals. This allowed the relative rank and impact of each website and journal to be assessed in their respective category.

We calculcated descriptive statistics for the website SMi and each of its components. We determined its temporal stability by correlating its values at one time point with its values one week, two weeks, three weeks, and seven months later. We also determined the correlation between the SMi on December 29, 2013, and the age of each website.

We measured the correlation between traditional journal impact metrics (Journal impact factor, Five-year journal impact factor, Immediacy index, Cited half-life, Eigenfactor, and Article influence score), the journal SMi score, and the components of the journal SMi (Google PageRank, Alexa Rank, Twitter Followers, and Facebook Likes). Spearman rank correlations were used for the analysis due to the non-linear monotonic associations present in the data. We used a two-sided alpha of 0.05 to determine statistical significance.

## RESULTS

One hundred sixty-three of 245 (66.5%) of the websites and 29 of 44 (65.9%) of the journals met the outlined inclusion criteria. The mean (SD) and median (IQR) age of EMCC websites was 2.9 (1.9) years and 2.0 (2.0) years with the oldest being 12 years old.

### SMi Derivation

We assessed five selected variables for inclusion in the SMi, but Google+ was excluded because few (6.7%) of the websites had substantive accounts (>100 followers). Substantive accounts were available for a much greater proportion of websites on Alexa (95.7% ranked), PageRank (76.7% rated >0), Twitter (71.8% had >100 followers) and Facebook (25.2% had >100 likes).

The formulas that we considered are listed below where A=Alexa; P=PageRank; T=Twitter; F=Facebook; x=blog, podcast, or journal; m=maximum value; Rx= rank of x (Figure). The four components were given equal weight by normalizing the values on a scale of 0 to 2.5 to produce a total website SMi or journal SMi with a minimum score of 0 and maximum score of 10.

Although the logarithmic and ordinal versions of the SMi were highly correlated (Spearman r>0.95), the logarithmic version of the SMi (logSMi) was judged to have the best operational characteristics because it was the most normally distributed and least subject to skewness of the individual components. Therefore, it was selected as the definitive SMi formula for further evaluation and henceforth will be referred to as the SMi.

### Temporal Characteristics

The SMi was significantly correlated with website age (r=0.372, p-value<0.001) and itself over one-week, two-week, three-week, and seven-month periods:

December 29, 2013 to January 5, 2014, r=0.991, p-value<0.001; December 29, 2013 to January 12, 2014, r=0.796, p-value<0.001; December 29, 2013 to January 19, 2014, r=0.806, p-value<0.001; December 29, 2013 to July 27, 2014, r=0.929, p-value<0.001.

### Social Media Followership

The SMi demonstrated a wide range with normal distribution. For websites the mean (SD) was 4.52 (1.65), with a range from 1.06 to 9.40. When applied to the included journals the SMi had a mean (SD) of 6.27 (1.30) with a range from 3.84 to 9.26.

Social media followership for websites and journals varied widely across each component of the SMi (Table 2). ECG Experts Study Cards (112,696 Facebook followers) and Life in the Fast Lane (14,216 Twitter followers) had high social medial followership for websites while the *New England Journal of Medicine* (847,603 Facebook followers) and *JAMA* (350,000 Twitter followers) had high social media followership for journals.

Ranked in their own media categories by SMi (Table 3a and 3b), the top three websites were Life in the Fast Lane (9.40), Academic Life in Emergency Medicine (8.89), and EMCrit (8.68). The top three journals (Table 3) were *New England Journal of Medicine* (9.26), *British Medical Journal* (9.09), and *JAMA* (8.75). The large increase in SMi, by approximately one standard deviation, between *American Journal of Critical Care* and *Lancet* illustrates the jump from specialty-specific EMCC journals to general medical journals. The highest ranked emergency medicine-specific journals were *Annals of Emergency Medicine* (6.61), *Emergency Medicine Journal* (6.22), and *Academic Emergency Medicine* (5.96)*.*

### Correlation with Journal Impact Factors

Traditional journal impact metrics correlated significantly with journal SMi score (Table 4). The strongest correlations were seen between the journal SMi score and Immediacy Index (r=0.609, p-value=<0.001) and Article Influence Score (r=0.608, p-value<0.001). Five-year Journal Impact Factor (r=0.526, p-value=0.001), Journal Impact Factor (r=0.526, p-value=0.003), and the Eigenfactor score (r=0.425, p-value=0.02) correlated less strongly.

When assessed alone, each of the journal SMi components also correlated with traditional journal impact metrics (Table 4). This was particularly true for Alexa Rank and Google PageRank, which correlated more strongly than the journal SMi in several cases.

## DISCUSSION

Regardless of one’s beliefs in the merit of using secondary sources such as blogs and podcasts for medical education, their rapid growth^1^ and surveys of medical learners^23^,^24^ suggest that they are increasingly being created and used. We developed the SMi score as a first step to identify a metric to assess the quality of social media-based educational resources, because such a gold standard currently does not exist. As an indirect measure of quality, we identified online measures of impact based on four followership variables, similar to how journals historically use impact measures as a surrogate for quality in the academic world.^11^,^12^

The SMi has several characteristics that make it a viable measurement of impact for learners, educators, and administrators. First, learners, educators, and administrators can apply these publically available metrics and transparent SMi formula without permission or cost. Second, our assessments of the SMi’s temporal attributes suggest that it measures long-term impact, rather than spikes in popularity. Furthermore, it is not unduly influenced by longevity, suggesting it is possible for new resources to be recognized.

Because no gold standard exists to measure social media educational resource impact, we examined how the SMi formula for journal websites would perform in comparison to traditionally recognized journal impact metrics. Our data found that a journals’ online followership, as quantified by the SMi formula, correlates with these metrics. Its particularly strong correlation with the Immediacy Index^25^ and Article Influence Score^9^,^10^ suggests that in journals it is most predictive of fast citations and influential articles. Further optimization of the SMi by weighting its components based on their correlation with journal impact was not performed because (1) no single gold standard exists for journal impact and (2) the impact of educational websites and journals may not correlate perfectly with the impact of journals.

Two of the four components of the SMi, Alexa Rank and Google PageRank, focus on website traffic and inbound links.^16^,^17^ As higher-impact journals are likely to have higher traffic webpages and a greater number of inbound links, it follows that these two web rankings correlated strongly with traditional measures of journal impact presumably because they publish articles that are discussed and read more frequently. However, to our knowledge this finding has not previously been reported in the literature. It may be of interest to journal publishers who would like to track their impact more closely.

The other two components of the SMi, Twitter Followers and Facebook Likes, also correlated with traditional journal impact factors. This is unsurprising as the altmetrics of individual articles have been shown to correlate with future citations,^15^ and journals with higher social media followership would be more likely to have their content shared. However, the correlations for Twitter Followers and Facebook Likes with journal impact factors were not as high as Alexa Rank and Google PageRank. Despite this, we believe Twitter Followers and Facebook Likes are important indicators to include within the SMi because they are likely better measures of followership, whereas Alexa and Google PageRank focus slightly more on viewership.^26^ We hypothesize that followership is an indirect measure of source credibility and thus an important measure of impact for these resources. While it is not a perfect parallel, following the social media accounts of a blog or podcast mirrors subscribing to a journal and is a significantly greater commitment than reading a single post, listening to a single podcast, or downloading a single journal article. For this reason we believe that the followership of social media channels, despite not correlating quite as well with journal impact, provides a different but important perspective on the impact of blogs or podcasts that would be lost were one of the other two metrics (Alexa or Google PageRank) considered alone.

To further the research agenda on the assessment of social media educational resources, our research group is in the process of deriving a quality assessment tool for blogs and podcasts using education literature and data from modified Delphi surveys of stakeholders. Future studies will assess the validity of this quality assessment tool and its correlation with the SMi. Our hypothesis that followership is a surrogate marker of quality will continue to be tested and modified with this research.

Moving forward, we are designing a program that will gather the required data, calculate the SMi, and update a webpage on a weekly basis. The results will be openly accessible on the website http://aliem.com/social-media-index. Additionally, as online resources are developed outside of EMCC we anticipate calculating rankings for medical education blogs and podcasts in other health professions.

## LIMITATIONS

Whenever an evaluation tool is developed that openly defines the individually measured components, it becomes possible to ‘game’ the system.^27^ The ability of the SMi to assess impact would be compromised if websites attempted to influence their scores by purchasing fictional followers and web traffic. This underhanded and artificial means to boost analytics numbers, however, would sabotage the professional credibility and reputation of the website owners. The tremendous risk of losing reader/listener trust and respect, along with the associated costs, would likely sway these volunteer websites away from manipulating such metrics. Notably, this limitation is not exclusive to the SMi as gaming has been a strong criticism of traditional impact metrics through self-referencing and preferential article publication/classification.^28^,^29^

There are many other social media platforms used by blogs and podcasts that were excluded from the SMi. Not taking these platforms into account may underscore websites that use platforms such as Google+, YouTube, and iTunes to distribute their content. However, due to the small number of websites using these platforms (Google+ and YouTube) and lack of publicly available metrics (iTunes) they were excluded from the current iteration of the SMi. As social media continues to evolve, the SMi may be modified to accommodate trends in its use.

In this study the SMi was derived using a subpopulation of medical education websites (blogs and podcasts) focused on a relatively specific field (EMCC). This was done intentionally to provide a homogenous group of websites for derivation of the SMi. However, its generalizability would be strengthened if it were applied successfully to other online educational products from various fields of medicine. Follow-up studies using the methodology outlined in this study and websites/journals from other specialties could provide further validity evidence for the SMi.

The selection of time intervals to assess the temporal stability of the SMi was somewhat arbitrary. We intended to demonstrate short-term stability with the weekly intervals and medium-term stability with follow-up approximately six months later; however, other time intervals could have been selected. We cannot speculate as to how this would have affected our results. While the collection of our final data point was slightly delayed, the strong week-to-week correlation at the beginning of the study suggests it would have been unlikely to change our results.

## CONCLUSION

The number of educational websites continues to grow, especially in the field of EMCC. The SMi has the potential to be a stable and accessible indicator of their impact. If the results of this study can be replicated it would benefit medical professionals by identifying resources for learners and assessing scholarly impact of educators that are using these media. Regardless of whether the SMi becomes the gold standard for the assessment of impact for online medical education resources, it should contribute to the discussion towards the development and validation of impact and quality metrics.

## Figures and Tables

**Figure:**
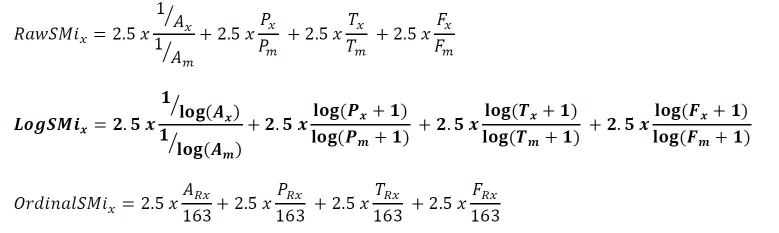
Formulas used for Social Media index (SMi) calculation.

**Table 1:** Definitions of the variables considered for the Social Media index.

	Website variable	Medical journal variables	Collection methodology
Alexa Rank	Alexa Rank of the blog/podcast website divided by 1000.	Alexa Rank or the website of the journal^a^ or the journal’s sponsoring organization^b^ (whichever is greater) divided by 1000. Journal pages on publisher’s websites were not used.	Alexa data was obtained using the Chrome SEO Status Toolbar^30^ and confirmed using Alexa.com.
Google PageRank	PageRank of the blog/podcast website.	PageRank of the journal website.	Google PageRank data was obtained using the Chrome SEO Status Toolbar^30^ and confirmed using the website CheckPageRank.net.
Twitter Followers	The number of followers of a contributor^c^ or website handle (whichever is greater).	The number of followers of a journal or sponsoring organization^b^ (whichever is greater).	Twitter follower data were obtained directly from the identified Twitter profile page.
Facebook Likes	The number of likes for the blog/podcast page.	The number of likes for the journal or sponsoring organization b page (whichever is greater).	Facebook like data were obtained directly from the identified Facebook page.
Google + members /followers	The number of website community members or followers (whichever is greater).	The number of journal or sponsoring organization^b^ community members or followers (whichever is greater).	Google+ members or followers data were obtained directly from the identified Google+ page.

a The Alexa Ranks for Journal websites that were part of a publisher’s website were not used as they represented the Alexa Rank of multiple journals.

b A medical organization listed as an official sponsor on the About page of the Journal.

c An author or editor listed on the Author or About page of the website.

**Table 2:** Summary statistics for the Social Media index and their individual components (163 websites and 29 journals).

	Minimum	Maximum	Median (IQR)	Mean (SD)
Websites (Jan 19, 2014)				
Alexa Rank	62	22300	5476 (6978)	7090 (5733)
Facebook Likes	0	112696	0 (120)	1407 (9614)
Google PageRank	0	5	2.0 (2.0)	2.2 (1.5)
Twitter Followers	0	14216	410 (1113)	1135 (1892)
RawSMi	0.01	7.65	1.17 (0.95)	1.39 (1.10)
LogSMi	1.06	9.40	4.58 (2.17)	4.52 (1.65)
OrdinalSMi	0.21	9.82	3.87 (3.65)	4.22 (2.37)
Journals (Jan 20, 2014)				
Alexa Rank	11	11939	1554 (3862)	3378 (4135)
Facebook Likes	0	847603	984 (19516)	31515 (128371)
PageRank PageRank	3	8	5.0 (2.0)	5.30 (1.19)
Twitter Followers	0	350000	1303 (4664)	18741 (60116)
Journal LogSMi	3.84	9.26	6.22 (1.54)	6.27 (1.30)

*SMi,* Social Media index

**Table 3a:** The top five websites as calculated and by the Social Media index and its components.

Website Jan 19, 2014	Alexa Rank	Facebook Likes	Twitter Followers	SMi^c^
1st	LITFL^b^ (62)	ECG Experts Study Cards (112696)	LITFL^b^ (14216)	LITFL^b^ (9.40)
2nd	ALiEM^a^ (140)	EMS 12 lead (45080)	EMCrit (9213)	ALiEM^a^ (8.89)
3rd	EMCrit (219)	ALiEM^a^ (15591)	EMS 12 lead (7144)	EMCrit (8.68)
4th	Don’t forget the bubbles (292)	Dr. Smith’s ECG Blog (10259)	RAGE podcast (6413)	EMS 12 lead (8.52)
5th	PedEM Morsels (492)	ImpactED Nurse (7356)	iTeachEM (6413)	ImpactED Nurse (7.65)

The numbers in parentheses indicate the raw value for each website or journal. PageRank values excluded from the table due to ties (only integer values from 0 to 10 are available).

a ALiEM, Academic Life in Emergency Medicine

b LITFL, Life in the Fast Lane

c SMi, logarithmic formula for the Social Media index

**Table 3b:** The top five journals as calculated by the Social Media index and its components.

Journal Jan 20, 2014	Alexa Rank	Facebook Likes	Twitter Followers	Journal LogSMi^g^
1st	BMJ^b^ (11)	NEJM^g^ (847603)	JAMA^d^ (350000)	NEJM^e^ (9.26)
2nd	NEJM^e^ (23)	SJTREM^f^ (121000)	NEJM^e^ (162000)	BMJ^b^ (9.09)
3rd	JAMA^c^ (28)	JAMA^c^ (86938)	BMJ^c^ (104000)	JAMA^c^ (8.75)
4th	Lancet (47)	Lancet (72074)	Lancet (101000)	Lancet (8.23)
5th	PLOS^d^ Med (179)	CHEST (39177)	PLOS^d^ Med (23000)	AJCC^a^ (6.89)

The numbers in parentheses indicate the raw value for each website or journal. PageRank values excluded from the table due to ties (only integer values from 0 to 10 are available).

a AJCC, American Journal of Critical Care

b BMJ, British Medical Journal

c JAMA, Journal of the American Medical Association

d PLOS, Public Library of Science

e NEJM, New England Journal of Medicine

f SJTREM, Scandinavian Journal of Trauma, Resuscitation and Emergency Medicine

g SMi, logarithmic formula for the Social Media index

**Table 4:** Spearman’s correlation of the journal Social Media index (January 20, 2014) and its components with traditional journal impact metrics^22^ (n=29 journals).

	SMi		Google PageRank		Alexa Rank		Twitter Followers		Facebook Likes	
					
Journal metric	r	p-value	r	p-value	r	p-value	r	p-value	r	p-value
Immediacy index	0.609	0.0006	0.603	0.0007	0.731	<0.0001	0.492	0.008	0.515	0.005
Article influence score	0.608	0.0008	0.693	<0.0001	0.708	<0.0001	0.494	0.009	0.512	0.006
Five-year journal impact factor	0.590	0.001	0.668	0.0001	0.692	<0.0001	0.466	0.01	0.503	0.007
Journal impact factor	0.526	0.003	0.572	0.001	0.647	0.0001	0.398	0.03	0.449	0.01
Eigenfactor	0.425	0.02	0.617	0.0004	0.577	0.001	0.336	0.07	0.284	0.14
Cited half-life	0.407	0.03	0.475	0.01	0.417	0.03	0.416	0.03	0.270	0.17
